# Genomic Predictors of Response to Metastasis-directed Therapy With or Without Androgen Deprivation Therapy

**DOI:** 10.1016/j.euo.2025.07.007

**Published:** 2025-09-20

**Authors:** Philip Sutera, Kim Van der Eecken, Yang Song, Amol C. Shetty, Elai Davicioni, James A. Proudfoot, Alexander Hakansson, Keara English, Jarey Wang, Ozan Cem Guler, Soha Bazyar, Sofie Verbeke, Jo Van Dorpe, Valérie Fonteyne, Bram De Laere, Lara Hathout, Ronald Ennis, Salma K. Jabbour, Biren Saraiya, Ryan Stephenson, Tina Mayer, Mark Mishra, Zaker Rana, Jason Molitoris, Ana Kiess, Daniel Y. Song, Theodore DeWeese, Kenneth J. Pienta, Phuoc T. Tran, Alejandro Berlin, Cem Onal, Piet Ost, Matthew P. Deek

**Affiliations:** a Department of Radiation Oncology and Molecular Radiation Sciences, Johns Hopkins University School of Medicine, Baltimore, MD, USA; b Departments of Pathology and Human Structure and Repair, Ghent University, Ghent, Belgium; c Institute for Genome Sciences, University of Maryland School of Medicine, Baltimore, MD, USA; d Department of Radiation Oncology, University of Maryland School of Medicine, Baltimore, MD, USA; e Veracyte, San Diego, CA, USA; f Department of Radiation Oncology, Baskent University, Ankara, Turkey; g Department of Pathology and Cancer Research Institute, Ghent University Hospital, Ghent, Belgium; h Department of Radiation Oncology, Ghent University Hospital, Belgium; i Department of Radiation Oncology, Rutgers Cancer Institute, Rutgers University, New Brunswick, NJ, USA; j Division of Medical Oncology, Rutgers Cancer Institute, Rutgers University, New Brunswick, NJ, USA; k Department of Oncology, Sidney Kimmel Comprehensive Cancer Center, Johns Hopkins University School of Medicine, Baltimore, MD, USA; l James Buchanan Brady Urological Institute, Johns Hopkins School of Medicine, Baltimore, MD, USA; m Princess Margaret Cancer Centre, University Health Network, Toronto, Canada

**Keywords:** Oligometastasis, Castration-sensitive prostate cancer, Metastasis-directed therapy, Genomics, Personalized medicine

## Abstract

Metastasis-directed therapy (MDT) is an emerging treatment option for metachronous oligometastatic castration-sensitive prostate cancer (omCSPC) and can delay time to progression and the need to initiate androgen deprivation therapy (ADT). However, optimal ways to synergize MDT and ADT are not known, and better personalization of MDT is needed. We examined the role of combined ADT and MDT and the ability of genomic alterations to provide prognostic and predictive information regarding response to MDT. We found that high-risk (HiRi) mutations in *TP53, BRCA1/2, ATM,* and *Rb1* are poor prognostic markers in omCSPC. In addition, patients harboring HiRi mutations experienced greater benefit from addition of ADT to MDT, indicating that these alterations are predictive biomarkers for treatment intensification. Our results suggest that genetic biomarkers might aid in treatment personalization for patients with omCSPC.

The spectrum theory of metastasis postulates some metastatic lesions behave more akin to locoregional than to systemic disease [1]. In line with this hypothesis, prospective trials of metastasis-directed therapy (MDT) in metachronous oligometastatic castration-sensitive prostate cancer (omCSPC) have demonstrated that stereotactic ablative radiation therapy (SABR) prolonged progression-free survival (PFS) and time to initiation of androgen deprivation therapy (ADT) [2–4].

ADT represents a standard of care in mCSPC. It remains unclear how to integrate combinations of MDT and ADT in metachronous omCSPC to synergize therapeutic efficacy and quality of life. Several studies suggest that high-risk (HiRi) mutations (*TP53, BRCA1/2, ATM,* and *RB1*) may provide prognostic or predictive information regarding treatment responses in omCSPC [2,5]. Thus, we investigated whether these HiRi mutations provide discriminative information regarding responses to MDT with or without ADT.

This was a retrospective analysis of a cohort of patients with omCSPC (≤5 lesions) from Johns Hopkins Hospital and Ghent University treated with SABR with or without a course of ADT, including those in the STOMP and ORIOLE trials (*n* = 70) [2–4]. The primary endpoint of interest was time to prostate-specific antigen (PSA) progression. Secondary endpoints were distant metastasis–free survival (DMFS), time to development of castration-resistant prostate cancer (T_dCRPC_) and overall survival (OS), as previously defined [2]. A HiRi mutational signature was defined as pathogenic alterations in *TP53, BRCA1/2, ATM,* and *RB1* identified via DNA panels (Foundation CDx and Tempus xT) [2,5]. A subset of 68 patients underwent RNA sequencing (RNA-seq; Tempus, Chicago, IL, USA). RNA-seq expression values for individual genes in the model were quantile matched to a cohort of prospectively collected samples with microarray expression data from the Genomics Resource for Intelligent Discovery (GRID) database using the *MatchIT* R package [6]. Quantile matched expression was analyzed using GRID software (Veracyte, San Diego, CA, USA). Signature scores, including Decipher and Androgen Receptor Activity (AR-A), were also analyzed.

Baseline characteristics were compared using the Wilcoxon rank-sum test for continuous variables, and Fisher’s exact test or a χ^2^ test for categorical variables. Survival analysis was performed using the Kaplan-Meier method, stratified by treatment type or high-risk mutation status, and compared using log-rank tests. Interaction terms were calculated for endpoints. All analyses were conducted using R (R Foundation for Statistical Computing, Vienna, Austria).

A total of 144 patients were included, of whom 91 received MDT alone and 53 received MDT + ADT. Median follow-up was 32.7 mo. Baseline characteristics are shown in Supplementary Table 1. Median ADT duration was 6 (range 1–24.3) mo. Concurrent ADT was associated with better median time to PSA progression (23.3 mo, 95% confidence interval [CI] 18–not reached [NR], vs 13.9 mo, 95% CI 11–17.2 without ADT; *p* = 0.03; Fig. 1A). ADT was similarly associated with longer median time to DMFS (25.8 mo, 95% CI 21.4–NR, vs 19 mo 95% CI 14.7–26.3 without ADT; p = 0.05; Fig. 1B). There were no significant differences in T_dCRPC_ or OS (Supplementary Fig. 1A, B).

A HiRi mutation was identified in 45 tumors (31.2%; Supplementary Table 2). Outcomes significantly differed by HiRi mutation status. ADT addition significantly prolonged the median time to PSA progression in the group of patients with HiRi mutations (23.3 mo, 95% CI 20.9– NR, vs 8.8 mo, 95% CI 6.8–12.1 without ADT; *p* < 0.001; Fig. 2A), but not in the group without a HiRi mutation (18.4 mo, 95% CI 16.8–NR, vs 17.2 mo, 95% CI 14.1–26.6 without ADT; *p* = 0.48, interaction *p* = 0.02; Fig. 2B). ADT improved median DMFS in the group with a HiRi mutation (25.8 mo, 95% CI 21.4–NR, vs 10.7 mo, 95% CI 5.6–19.0 without ADT; *p* = 0.003; Fig. 2C), but not in the group without a HiRi mutation (NR, 95% CI 18 mo–NR, vs 24.2 mo, 95% CI 17.1–30.8 without ADT; *p* = 0.31, interaction *p* = 0.24; Fig. 2D). Sensitivity analyses in which endpoints were measured from the end of MDT demonstrated similar results (Supplementary Fig. 2A–D). *PTEN* and *SPOP* mutations were not predictive for ADT benefit.

In the RNA-seq cohort, transcriptomic data stratified by high versus low Decipher and AR-A scores (dichotomized using the median) demonstrated similar trends. Specifically, tumors with higher Decipher and AR-A scores were associated with a greater benefit from addition of ADT to MDT (Supplementary Fig. 3A–H).

This study highlights the heterogeneity of treatment responses to MDT in omCSPC and suggests that biomarkers might aid in predicting treatment responses. Specifically, tumors with HiRi mutations appeared to benefit from treatment intensification involving ADT addition to MDT. Our previous study demonstrated benefits from MDT over observation [2], and the present findings appear to identify patients with more aggressive disease biology requiring treatment intensification. Thus, this biomarker may aid in better personalization of treatment for patients with omCSPC.

Several prospective trials have established the efficacy of local therapies in omCSPC and demonstrated that MDT prolongs time to ADT initiation and disease progression [2–4]. Thus, there is interest in understanding how to integrate MDT in omCSPC, especially in conjunction with ADT. One approach is a paradigm such as that used in the EXTEND or RADIOSA trials, which showed that addition of MDT to intermittent ADT improved eugonadal PFS in omCSPC [7,8]. An alternative approach is to identify biomarkers that can predict treatment responses. Results from our study suggest that HiRi mutations (*TP53, BRCA1/2, ATM,* and *RB1*) might provide predictive information regarding responses to MDT with or without ADT. Tumors harboring HiRi alterations benefited from addition of ADT to MDT, while those without HiRi alterations did not, so these HiRi mutations could have potential for identifying patients who could be managed with MDT alone and avoid the toxicity of ADT. A predictive ability of HiRi mutations has been demonstrated in other metastatic CSPC states [2,5] and future prospective trials to validate these findings are needed.

Our study has several limitation, including its retrospective nature (although many patients were treated in prospective studies), which opens the results to potential biases. In addition, genes of interest were not prespecified and were limited to those on available sequencing panels. Thus, these results are hypothesis-generating. Finally, ADT duration was not prespecified. However, the median duration was 6.1 mo with an interquartile range of 6–7.9 mo, so the duration was similar for most patients who received ADT.

In conclusion, HiRi mutations in *TP53*, *BRCA1*/2, *ATM*, and *RB1* appear to provide predictive information that may guide treatment intensification in omCSPC. Our results suggest that biomarkers could be used in the future to develop MDT paradigms.

## Supplementary Material

Supp Fig 1

Supp Fig 2

Supp Fig 3

Supp Fig 4

Supp Fig 6

Supp Fig 5

Supp Fig 7

Supp Fig 9

Supp Fig 8

Supp Fig 10

Supp Fig 11

Supp Fig 12

Supp Fig 13

Supp Fig 14

Supp Table2

Supp Table 1

## Figures and Tables

**Fig. 1 –:**
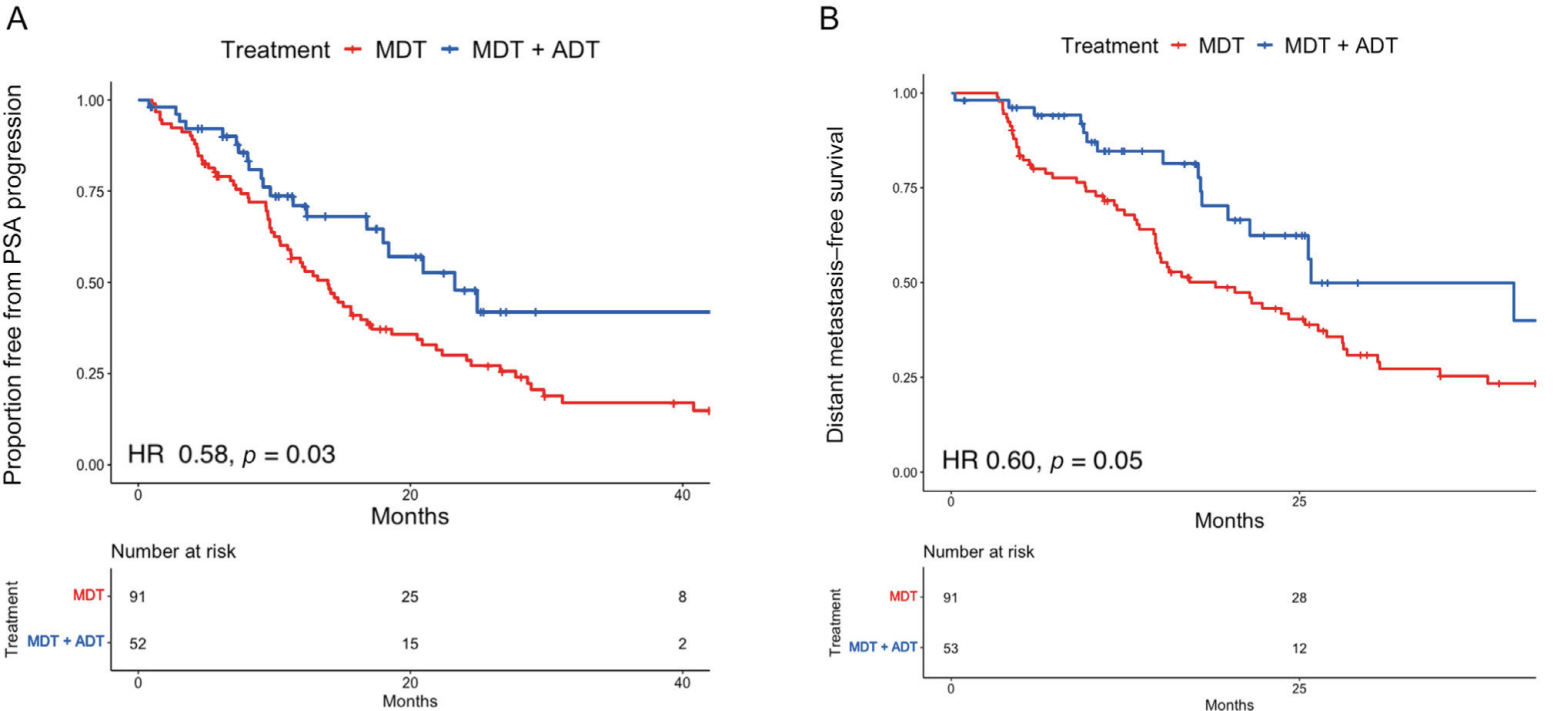
Kaplan-Meier plots of the risk of (A) PSA progression and (B) DMFS for the entire cohort, stratified by treatment arm. Concurrent ADT was associated with longer median time to PSA progression (23.3 mo, 95% CI 18–NR vs 13.9 mo, 95% CI 11–17.2; p = 0.03) and longer median DMFS (25.8 mo, 95% CI 21.4–NR vs 19 mo, 95% CI 14.7–26.3; *p* = 0.05). ADT = androgen deprivation therapy; CI = confidence interval; DMFS = distant metastasis–free survival; HR = hazard ratio; MDT = metastasis-directed therapy; NR = not reached; PSA = prostate-specific antigen.

**Fig. 2 –:**
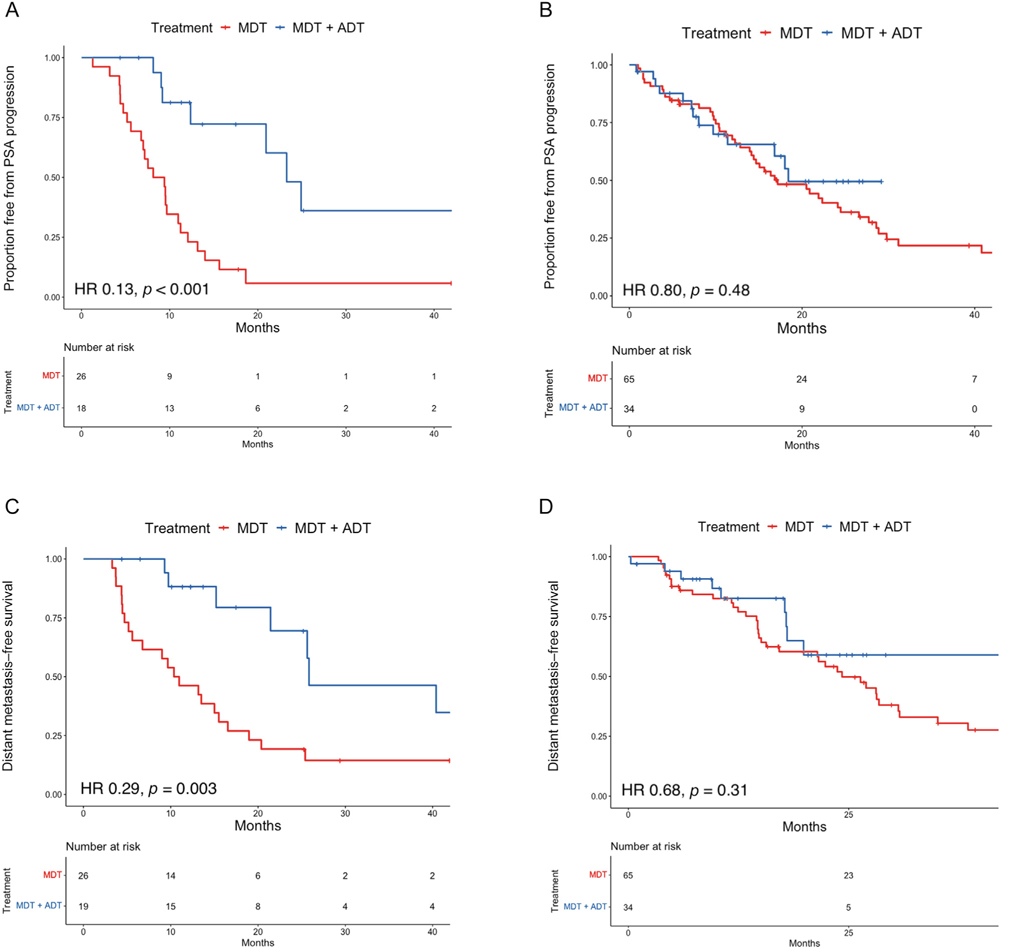
(A,B) Kaplan-Meier plots of the risk of PSA progression stratified by treatment arm for patients (A) with and (B) without HiRiMs. ADT addition significantly prolonged the median time to PSA progression in the group with HiRiMs (23.3 mo, 95% CI 20.9–NR vs 8.8 mo, 95% CI 6.8–12.1; *p* < 0.001), but not in the group without a HiRiM (18.4 mo, 95% CI 16.8–NR vs 17.2 mo, 95% CI 14.1–26.6; *p* = 0.48). (C,D) Kaplan-Meier plots of the risk of DMFS stratified by treatment arm for patients (C) with and (D) without HiRiMs. ADT addition improved median DMFS in the group with HiRiMs (25.8 mo, 95% CI 21.4–NR vs 10.7 mo, 95% CI 5.6–19.0; *p* = 0.003), but not in the group without a HiRiM (NR, 95% CI 18 mo–NR vs 24.2 mo, 95% CI 17.1–30.8; *p* = 0.31). ADT = androgen deprivation therapy; CI = confidence interval; DMFS = distant metastasis–free survival; HiRiMs = high-risk mutations; HR = hazard ratio; MDT = metastasis-directed therapy; NR = not reached; PSA = prostate-specific antigen.

## ADVANCING PRACTICE

### What does this study add?

Our study demonstrates that addition of androgen deviation therapy (ADT) to metastasis-directed therapy (MDT) prolongs the time to progression in metachronous oligometastatic castration-sensitive prostate cancer (omCSPC) and that genetic biomarkers appear to provide predictive information regarding response. Patients with alteration in high-risk genes (*TP53, BRCA1/2, ATM,* and *RB1*) experienced a significant benefit from ADT addition to MDT, but this benefit was not seen for patients without high-risk mutations. These findings suggest that patients with high-risk mutations might benefit from treatment intensification, while those without high-risk mutations could be managed with MDT alone and avoid the toxicity of ADT. Genomic alterations may provide information to guide treatment personalization in omCSPC.

### Clinical Relevance

Delaying Androgen deprivation therapy as much as possible could be relevant to diminishing treatment side effects and improvind QoL in oligometastatic castration sensitive prostate cancer, however, there may be some patients in which metastasis directed therapy (MDT) alone may not provide an adequate disease control. In this post hoc analysis of the ORIOLE and STOMP trials, suggest that patients whose tumors present detectable alterations in TP53, BRCA1/2, ATM and Rb1, associated with agressive disease, may benefit from the addition of ADT to MDT. These results build-up on the importance of considering tumor biology for treatment optimization in the CSPC scenario. Associate Editor: Elena Castro.

### Patient Summary

We looked at how tumor genetics can affect the response to radiotherapy and hormone therapy for patients with low-volume metastatic prostate cancer. We found that patients with mutations in genes associated with prostate cancer risk may benefit from more aggressive treatment.

## Appendix A. Supplementary data

Supplementary data to this article can be found online at https://doi.org/10.1016/j.euo.2025.07.007.
