# Supplementary figures and images for: Genomic Predictors of Response to Metastasis-directed Therapy With or Without Androgen Deprivation Therapy
Source: Eur Urol Oncol. Author manuscript; Available in PMC 2026 Jul 25. (PMC13401512; doi:10.1016/j.euo.2025.07.007)

# Development of Castration Resistance Stratified by Treatment Arm

Treatment + MDT + MDT + ADT

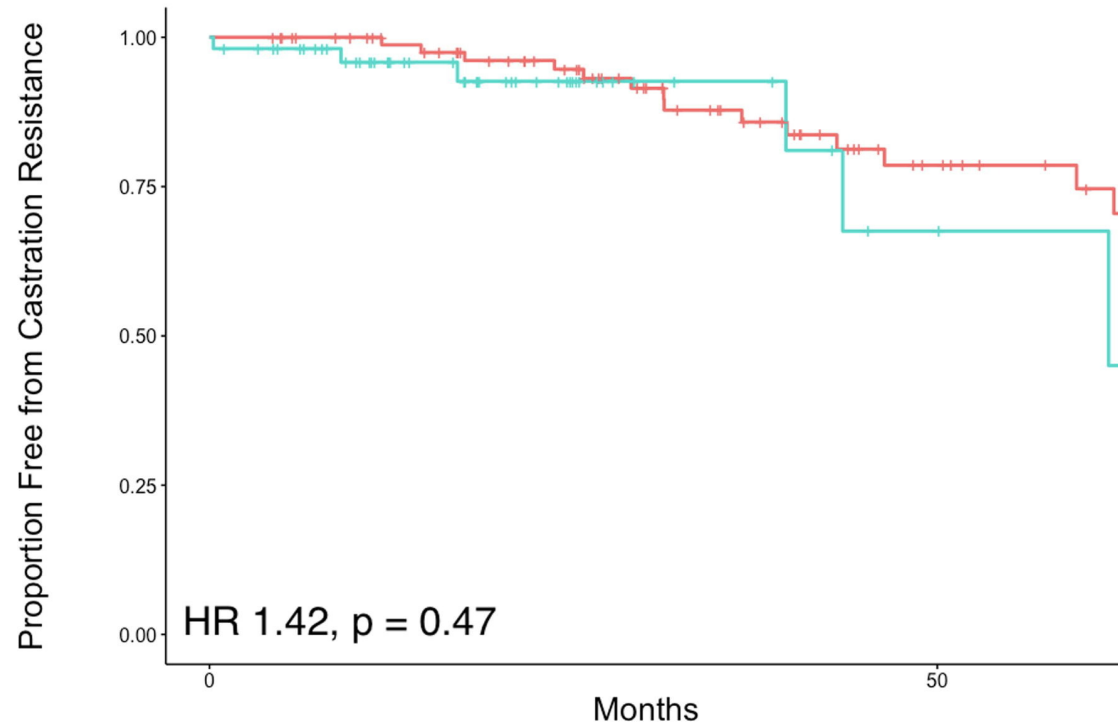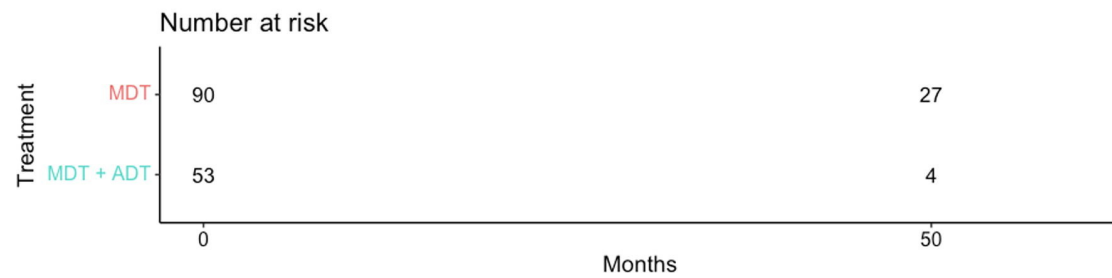

Supplement: Supp Fig 1 [file NIHMS2147580-supplement-Supp_Fig_1.pdf]

### Overall Survival Stratified by Treatment Arm

Treatment + MDT + MDT + ADT

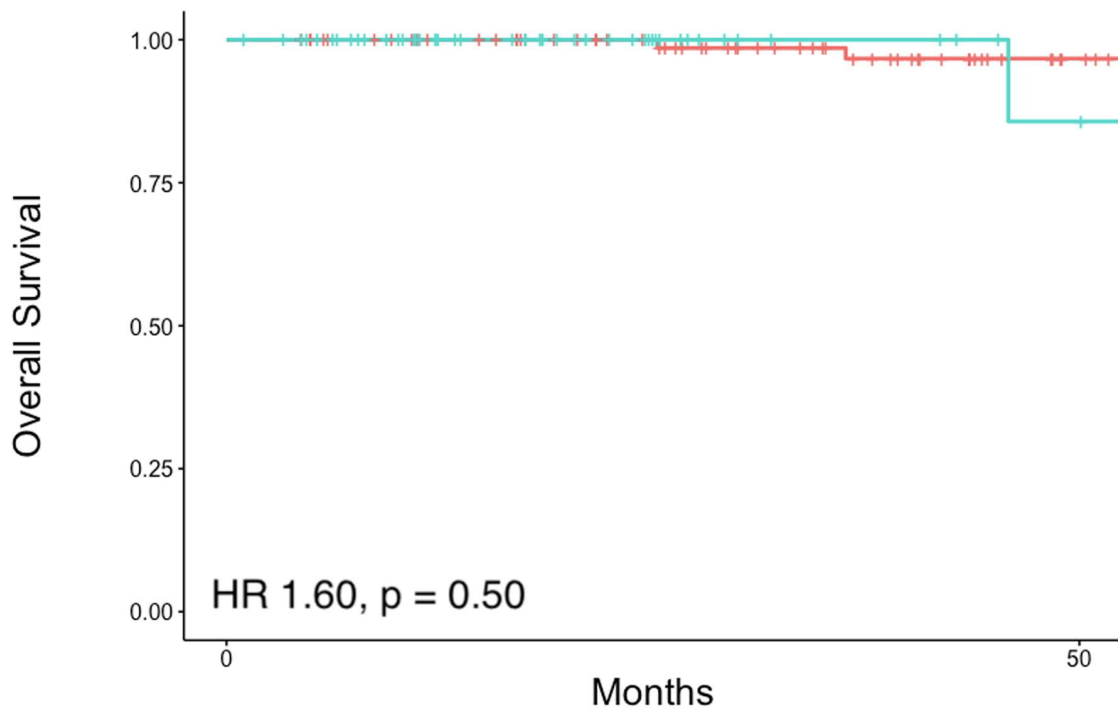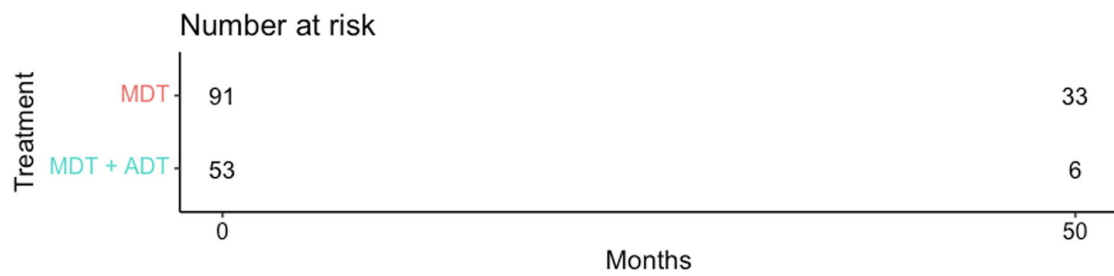

Supplement: Supp Fig 2 [file NIHMS2147580-supplement-Supp_Fig_2.pdf]

Rates of PSA Progression with High Risk Mutations

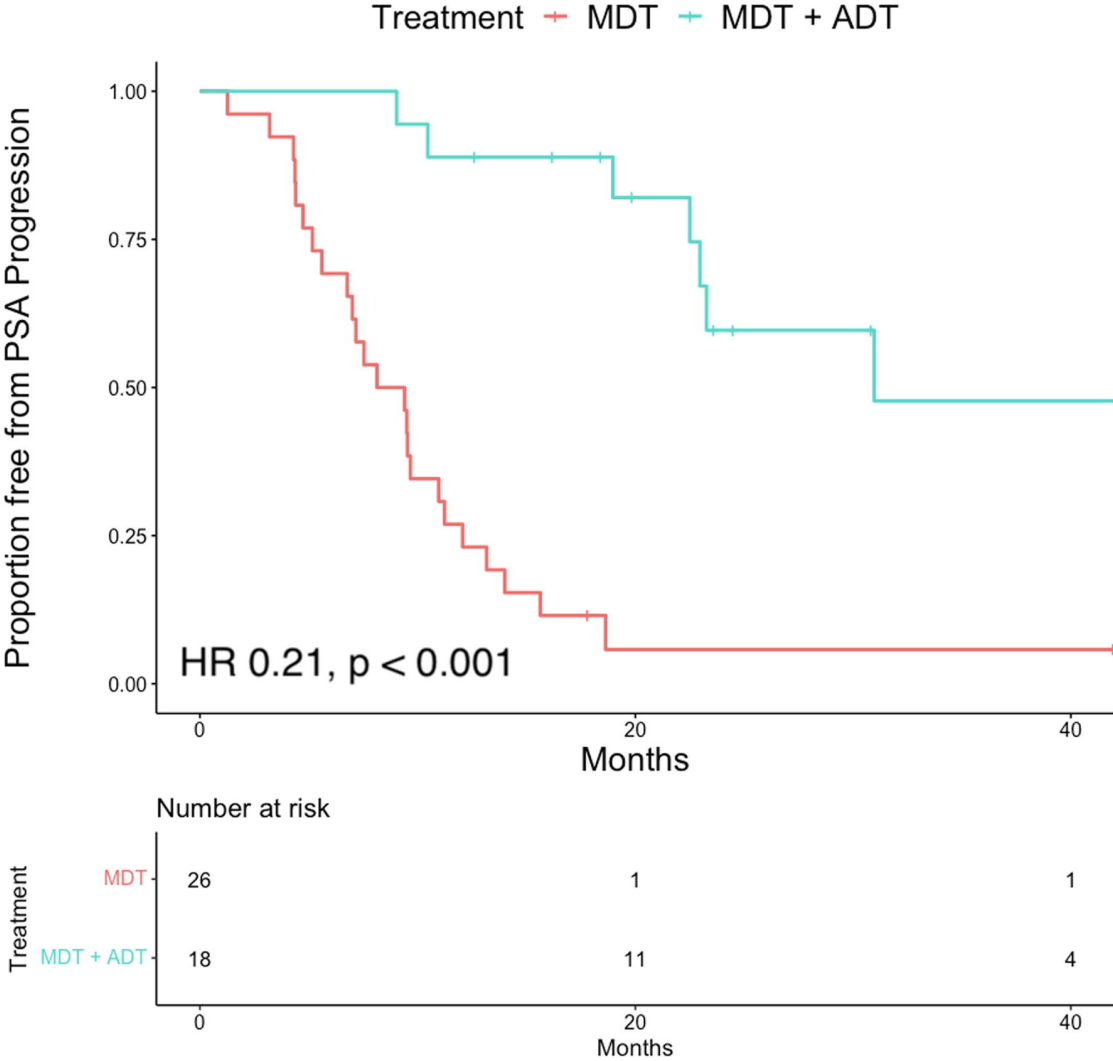

Supplement: Supp Fig 3 [file NIHMS2147580-supplement-Supp_Fig_3.pdf]

Distant Metastasis Free Survival without High Risk Mutations

Treatment + MDT + MDT + ADT

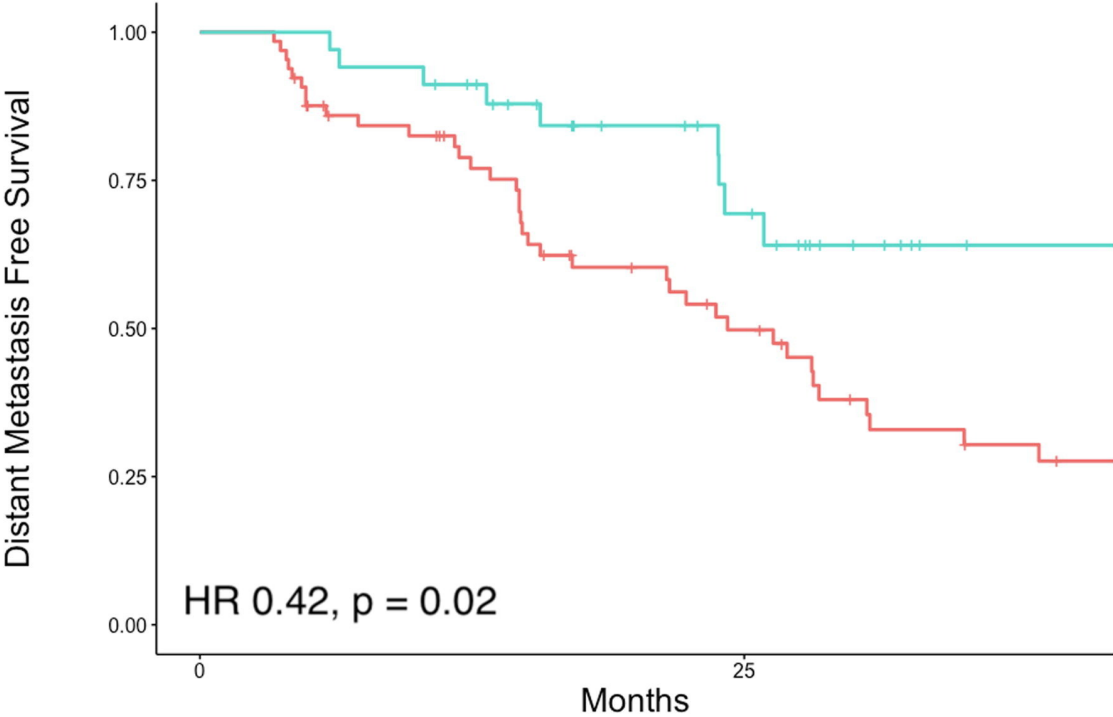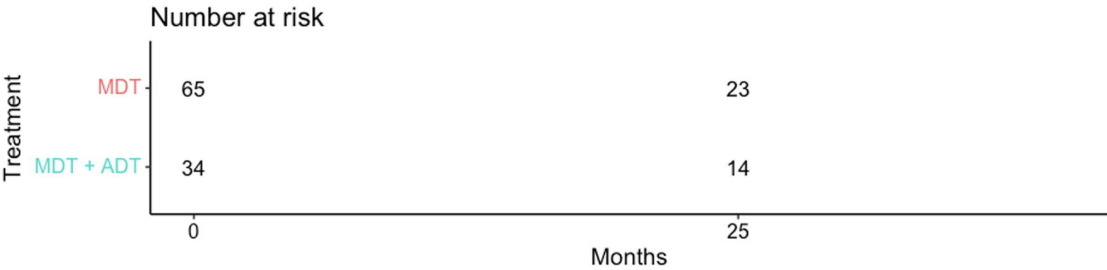

Supplement: Supp Fig 6 [file NIHMS2147580-supplement-Supp_Fig_6.pdf]

# Distant Metastasis Free Survival with High Decipher Score

Treatment + MDT + MDT + ADT

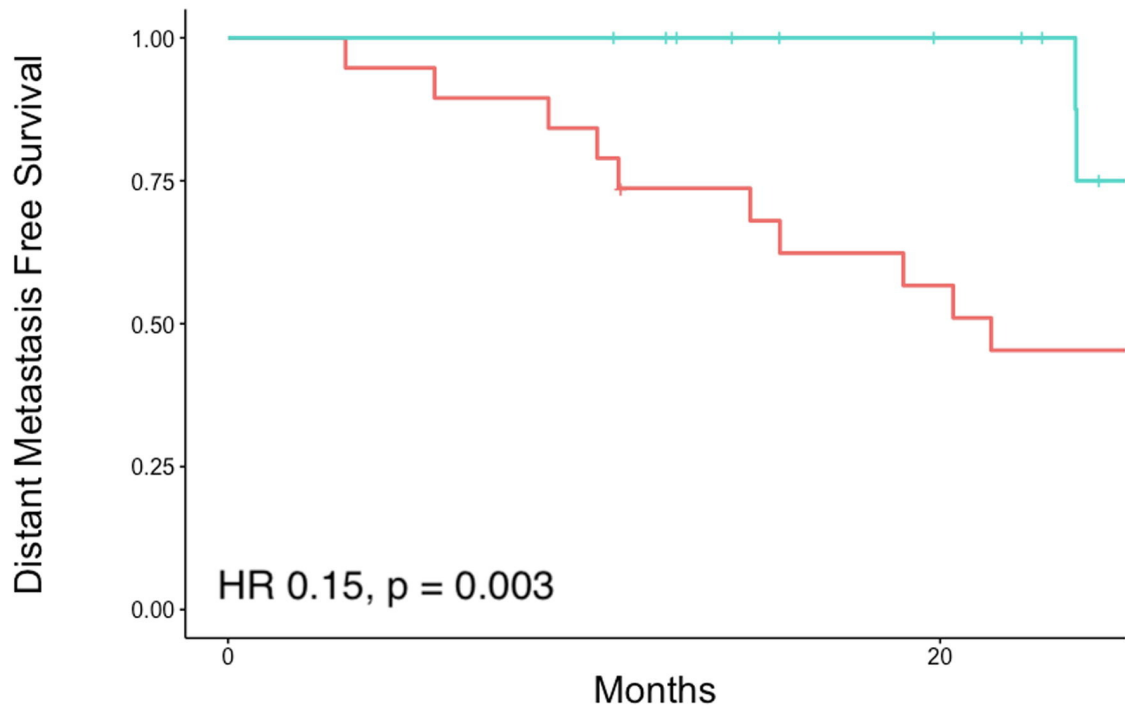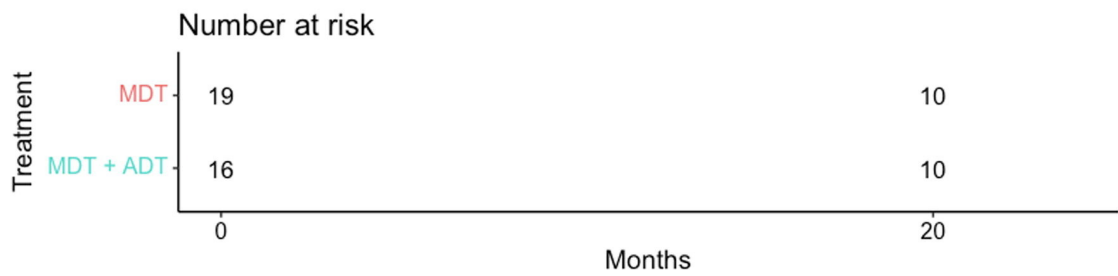

Supplement: Supp Fig 9 [file NIHMS2147580-supplement-Supp_Fig_9.pdf]

# Distant Metastasis Free Survival with Low Decipher Score

Treatment + MDT + MDT + ADT

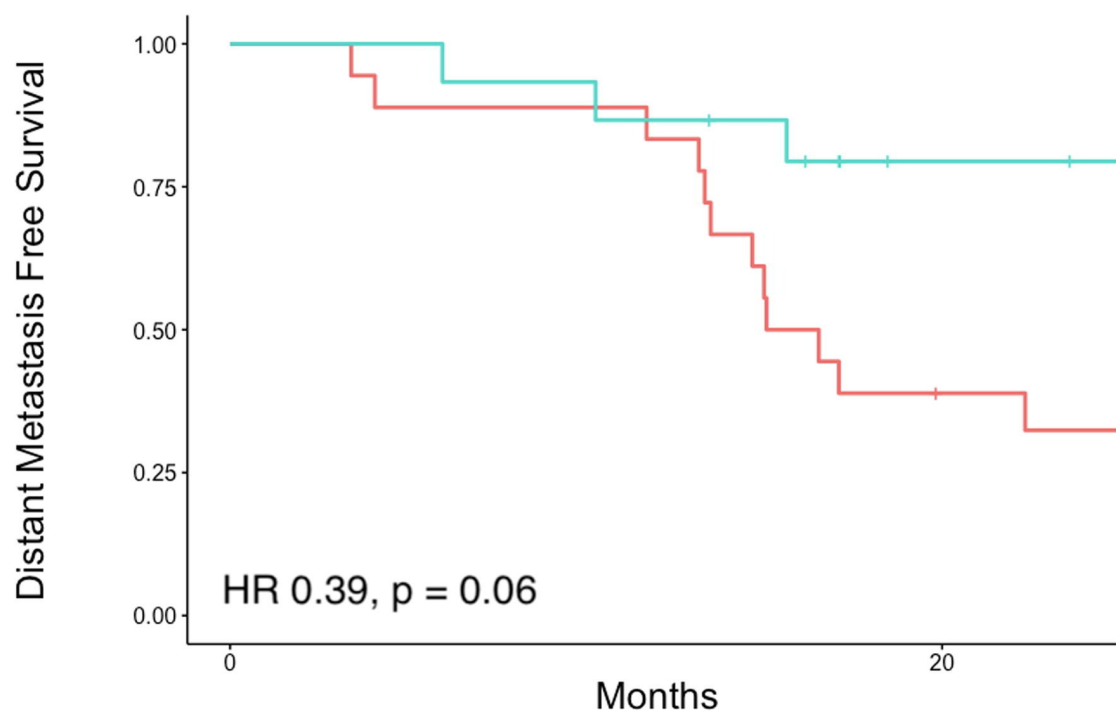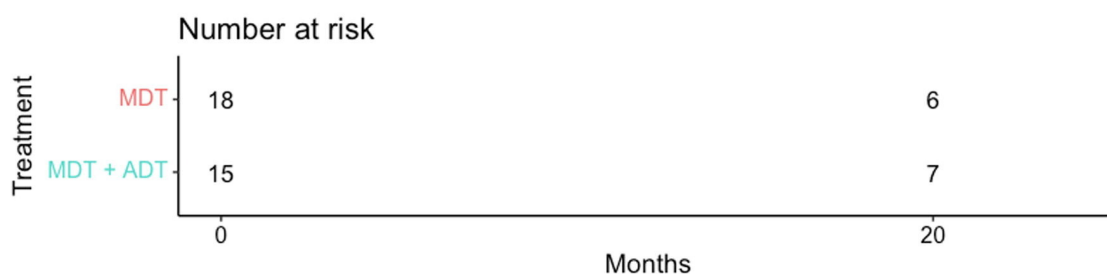

Supplement: Supp Fig 10 [file NIHMS2147580-supplement-Supp_Fig_10.pdf]

# Development of Castration Resistance Stratified by Treatment Arm

Treatment + MDT + MDT + ADT

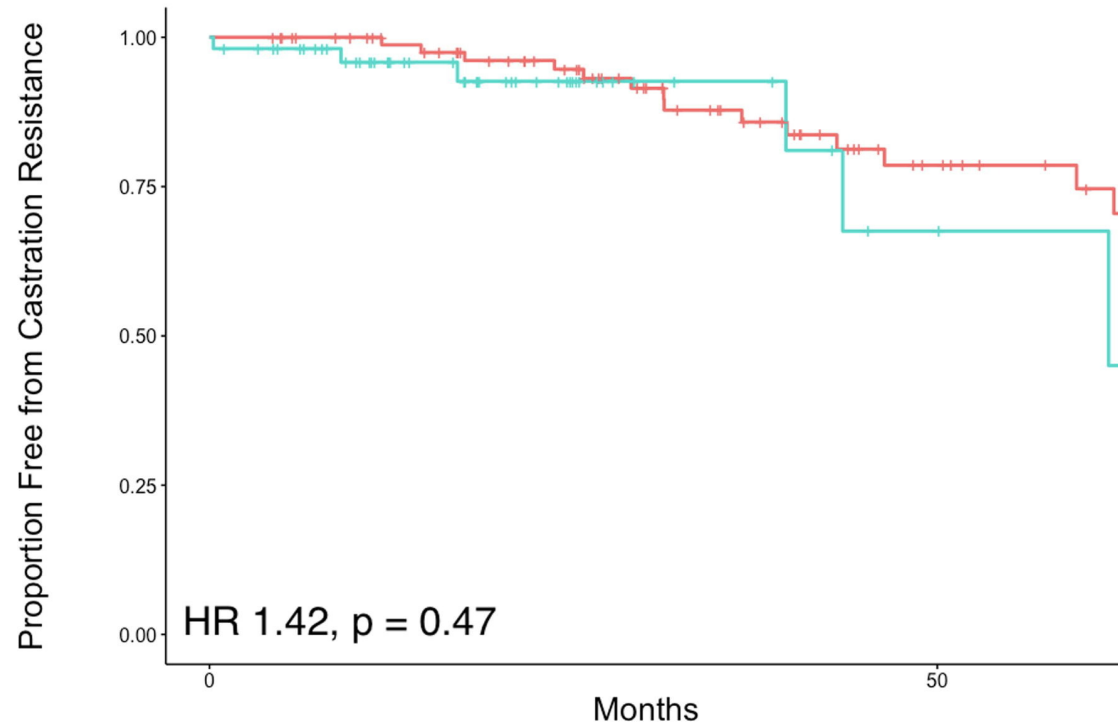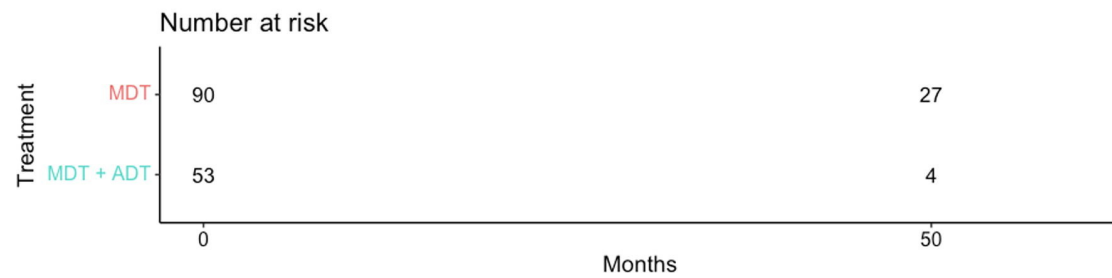

Supplement: Supp Table2 [file NIHMS2147580-supplement-Supp_Table2.pdf]

# Development of Castration Resistance Stratified by Treatment Arm

Treatment + MDT + MDT + ADT

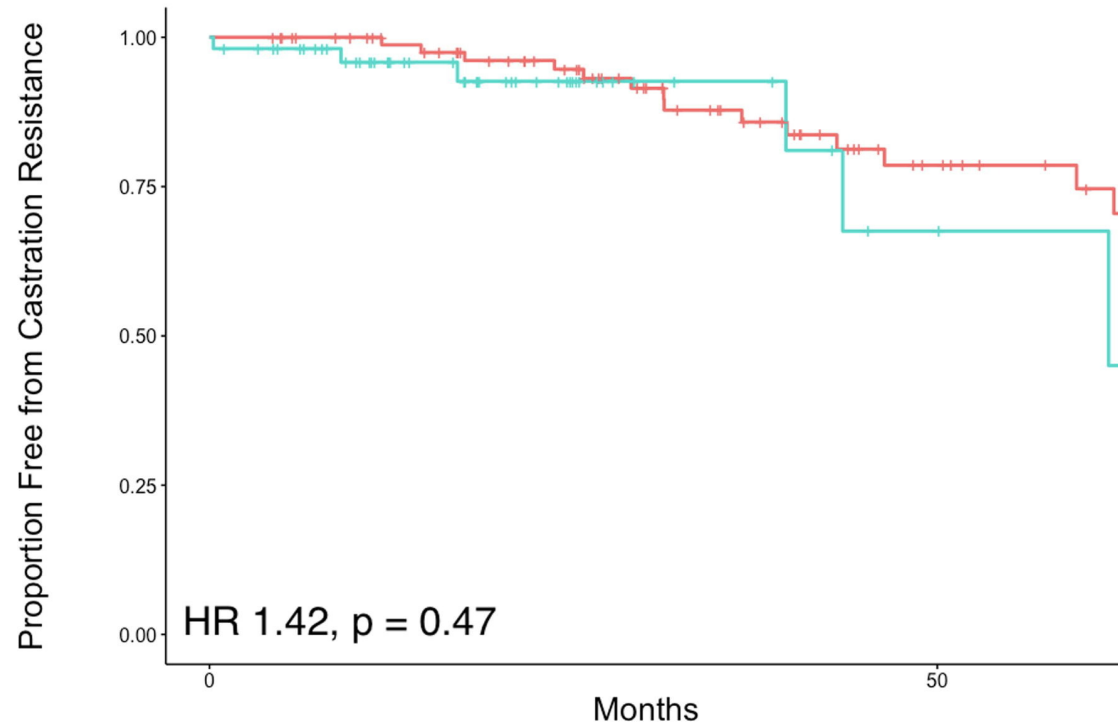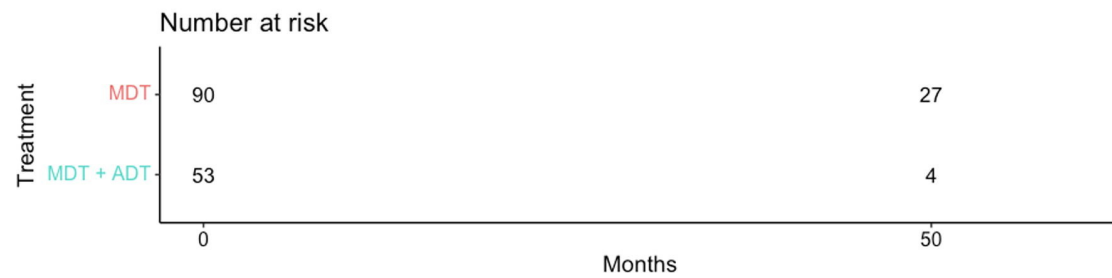

Supplement: Supp Table 1 [file NIHMS2147580-supplement-Supp_Table_1.pdf]
