# Supplementary material for: Genomic Predictors of Response to Metastasis-directed Therapy With or Without Androgen Deprivation Therapy
Source: Eur Urol Oncol. Author manuscript; Available in PMC 2026 Jul 25. (PMC13401512; doi:10.1016/j.euo.2025.07.007)
Supplement: Supp Fig 4 [file NIHMS2147580-supplement-Supp_Fig_4.pdf]

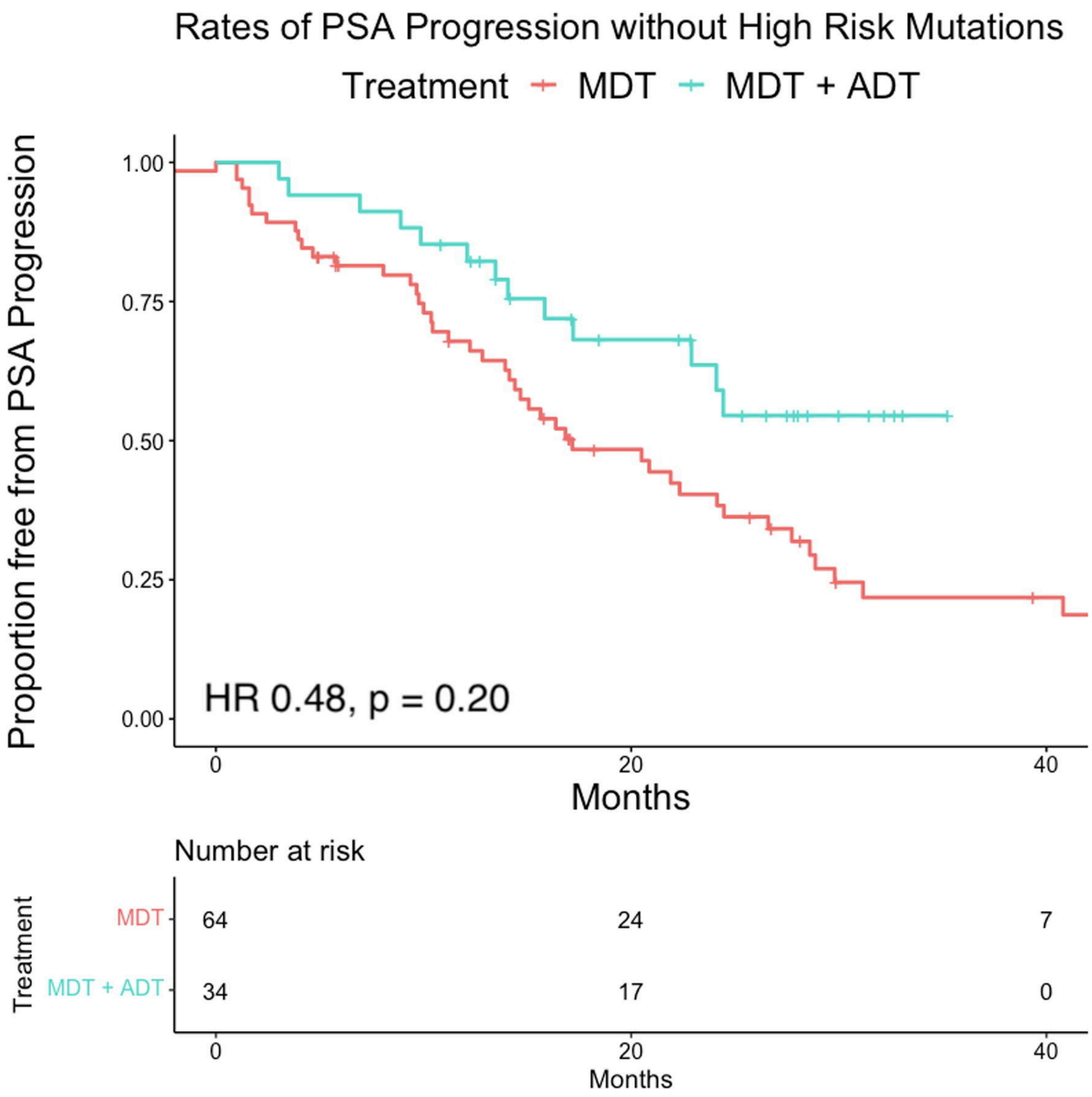
Rates of PSA Progression without High Risk Mutations
Treatment MDT MDT + ADT
Proportion free from PSA Progression
1.00
0.75
0.50
0.25
0.00
HR 0.48, p = 0.20
0
20
40
Months
Number at risk
Treatment
MDT 64 24 7
MDT + ADT 34 17 0
0
20
40
Months
