# Supplementary material for: Genomic Predictors of Response to Metastasis-directed Therapy With or Without Androgen Deprivation Therapy
Source: Eur Urol Oncol. Author manuscript; Available in PMC 2026 Jul 25. (PMC13401512; doi:10.1016/j.euo.2025.07.007)
Supplement: Supp Fig 5 [file NIHMS2147580-supplement-Supp_Fig_5.pdf]

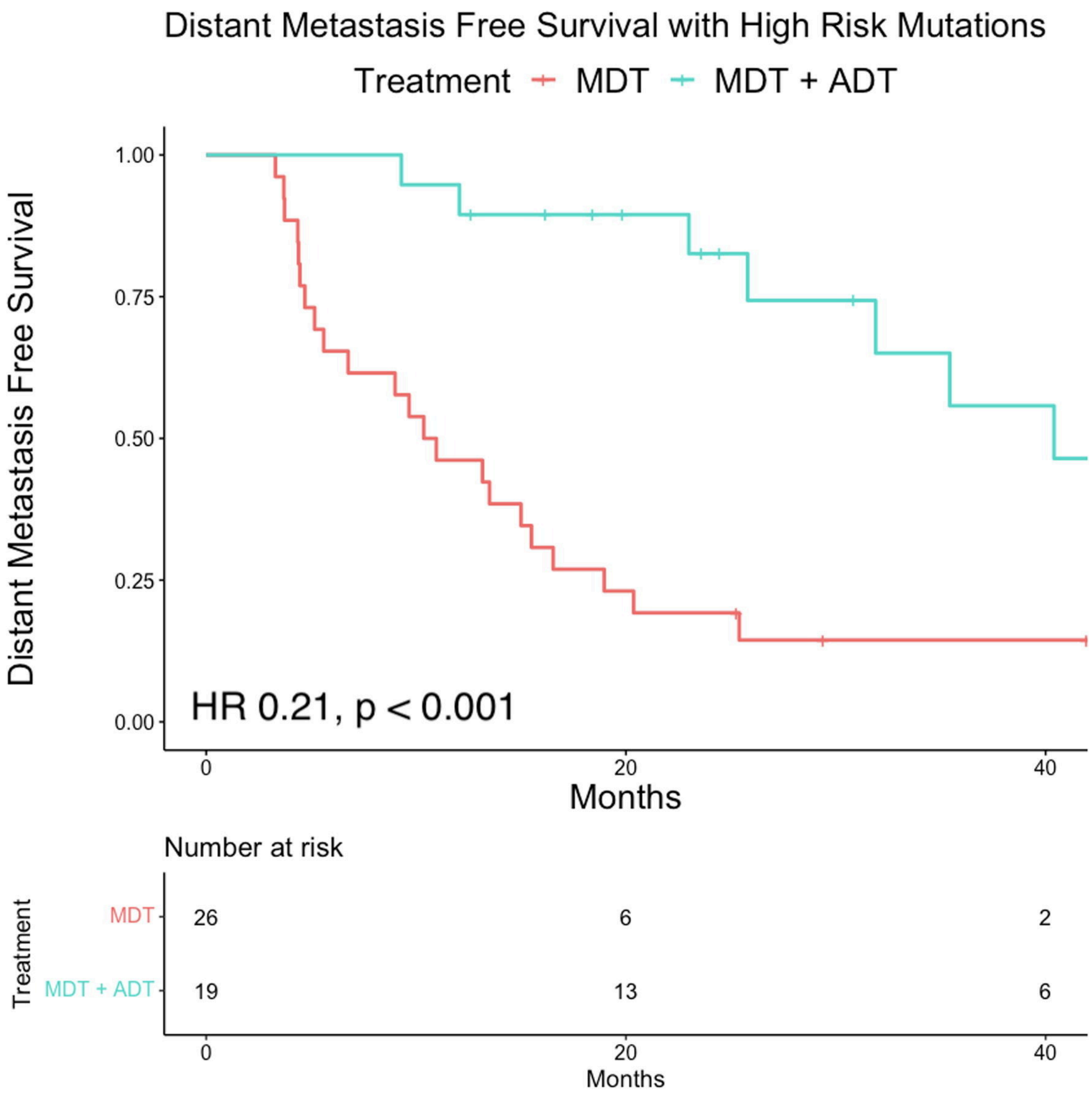
Distant Metastasis Free Survival with High Risk Mutations
Treatment
MDT
MDT + ADT
Distant Metastasis Free Survival
1.00
0.75
0.50
0.25
0.00
HR 0.21, p < 0.001
0
20
40
Months
Number at risk
Treatment
MDT
MDT + ADT
26
6
2
19
13
6
0
20
40
Months
