# Supplementary material for: Genomic Predictors of Response to Metastasis-directed Therapy With or Without Androgen Deprivation Therapy
Source: Eur Urol Oncol. Author manuscript; Available in PMC 2026 Jul 25. (PMC13401512; doi:10.1016/j.euo.2025.07.007)
Supplement: Supp Fig 11 [file NIHMS2147580-supplement-Supp_Fig_11.pdf]

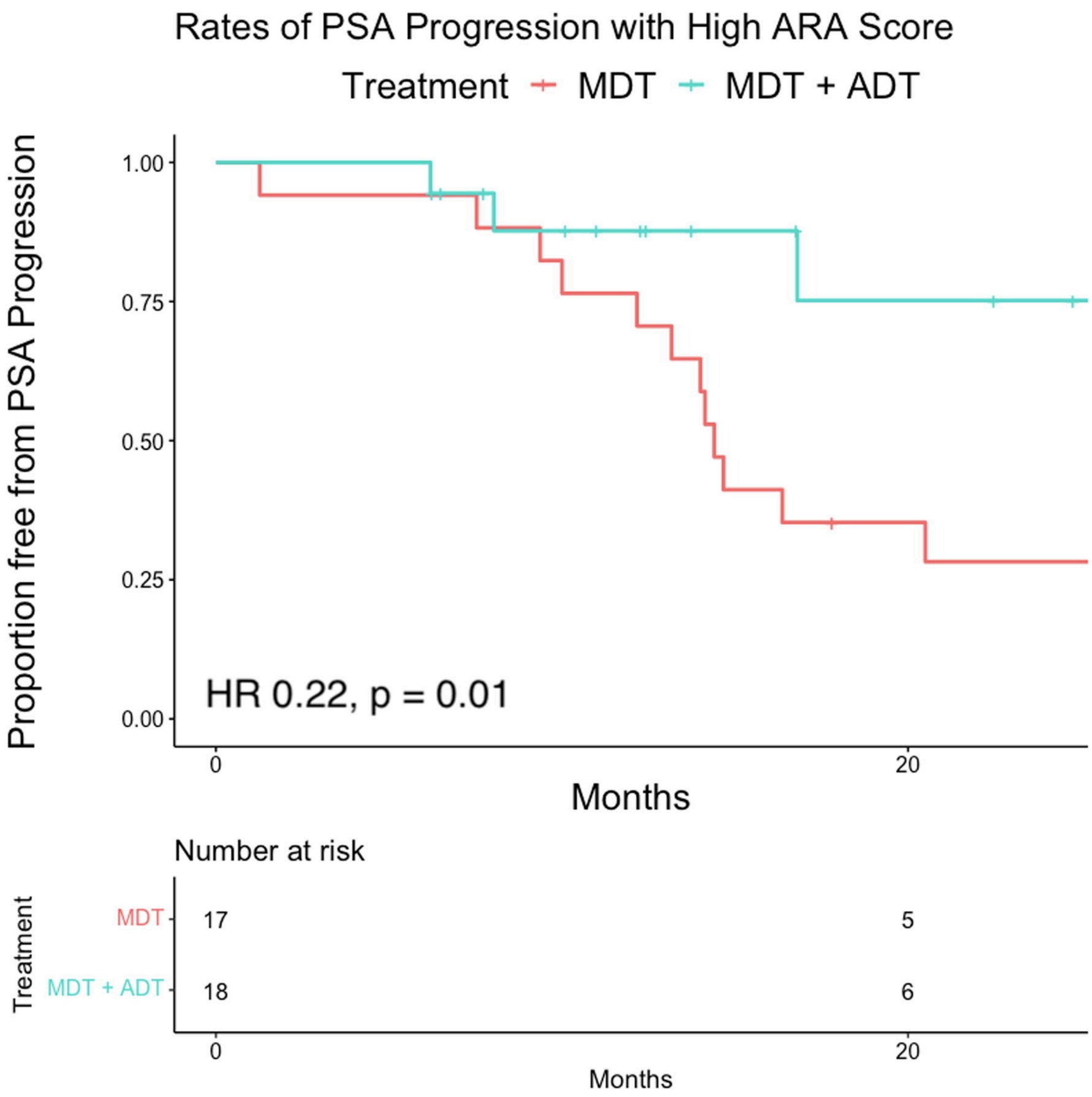

Rates of PSA Progression with High ARA Score
Treatment MDT MDT + ADT
Proportion free from PSA Progression
1.00
0.75
0.50
0.25
0.00
HR 0.22, p = 0.01
0
20
Months
Number at risk
Treatment
MDT 17 5
MDT + ADT 18 6
0
20
Months
