# Supplementary material for: Genomic Predictors of Response to Metastasis-directed Therapy With or Without Androgen Deprivation Therapy
Source: Eur Urol Oncol. Author manuscript; Available in PMC 2026 Jul 25. (PMC13401512; doi:10.1016/j.euo.2025.07.007)
Supplement: Supp Fig 12 [file NIHMS2147580-supplement-Supp_Fig_12.pdf]

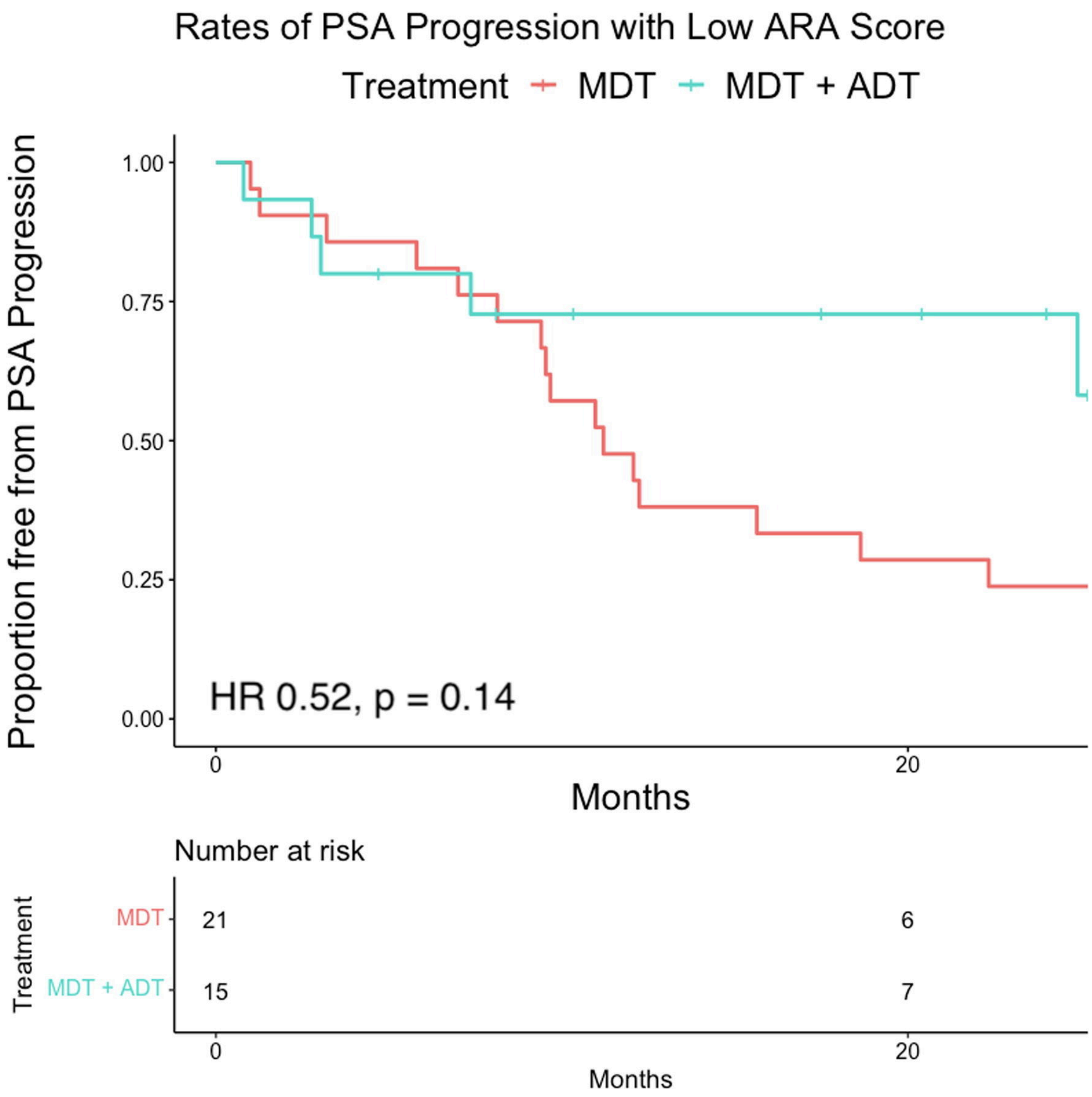

Rates of PSA Progression with Low ARA Score
Treatment MDT MDT + ADT
Proportion free from PSA Progression
1.00
0.75
0.50
0.25
0.00
HR 0.52, p = 0.14
0
20
Months
Number at risk
Treatment
MDT 21 6
MDT + ADT 15 7
0
20
Months
