# Supplementary material for: Genomic Predictors of Response to Metastasis-directed Therapy With or Without Androgen Deprivation Therapy
Source: Eur Urol Oncol. Author manuscript; Available in PMC 2026 Jul 25. (PMC13401512; doi:10.1016/j.euo.2025.07.007)
Supplement: Supp Fig 14 [file NIHMS2147580-supplement-Supp_Fig_14.pdf]

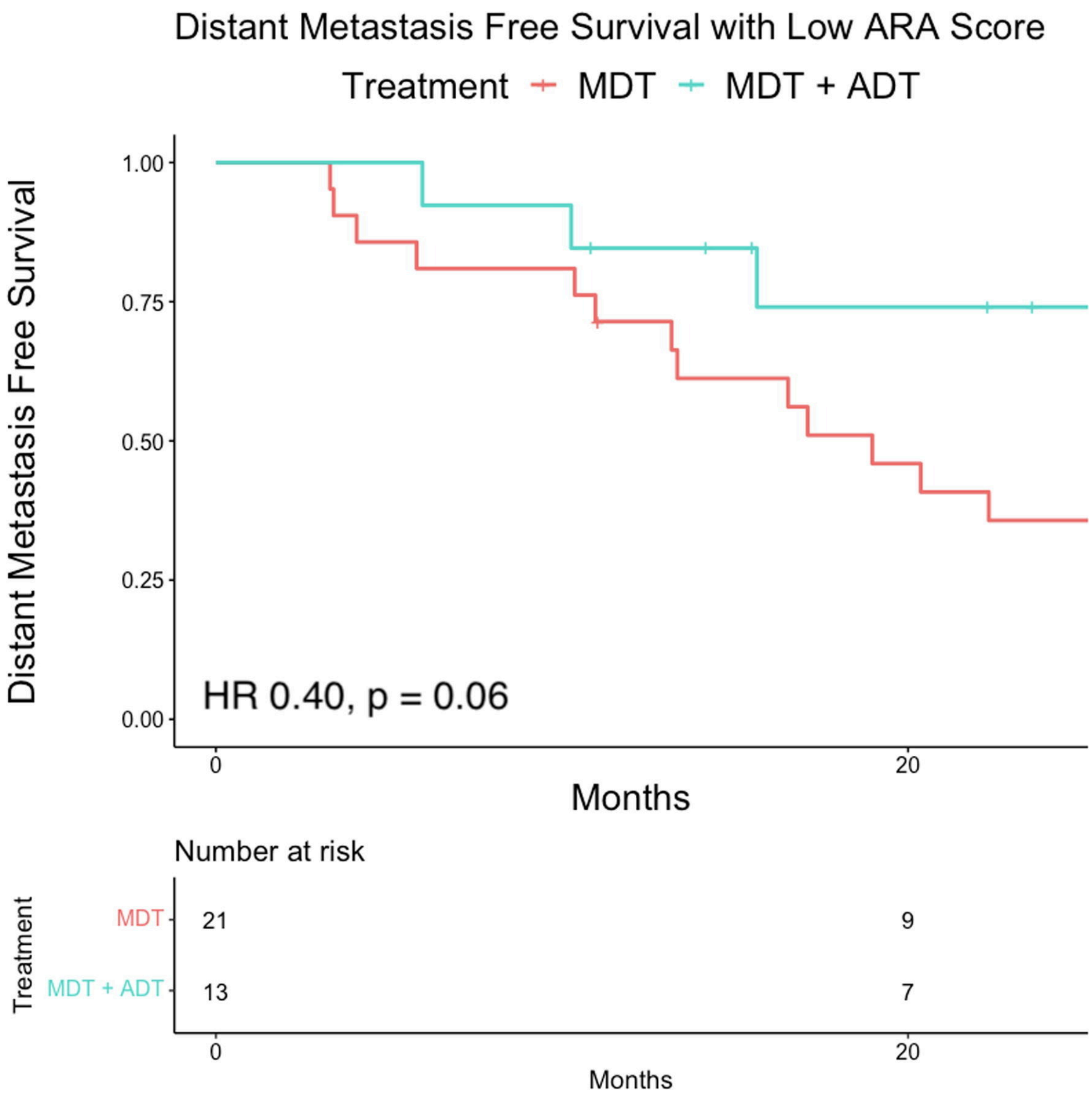
Distant Metastasis Free Survival with Low ARA Score
Treatment
MDT
MDT + ADT
Distant Metastasis Free Survival
1.00
0.75
0.50
0.25
0.00
HR 0.40, p = 0.06
0
20
Months
Number at risk
Treatment
MDT
MDT + ADT
21
9
13
7
0
20
Months
